# Toward Ambulatory Baroreflex Sensitivity: Comparison Between Indices of Arterial Line and Photoplethysmography in Male Volunteers

**DOI:** 10.2196/54771

**Published:** 2025-07-17

**Authors:** Jolanda Witteveen, Fabian Beutel, Evelien Hermeling

**Affiliations:** 1 One Planet Research Center, imec, Wageningen, The Netherlands; 2 imec The Netherlands/Holst Centre, High Tech Campus 31, Eindhoven, 5656 AE, The Netherlands

**Keywords:** photoplethysmography, pulse arrival time, pulse transit time, blood pressure, pulse wave analysis, heart rate, heart rate variability, baroreflex, arterial line, circadian rhythm, heart, arterial, arterial line, feasibility, systolic blood pressure, cyclic rhythm, feasibility test, baroreceptor sensitivity

## Abstract

**Background:**

Baroreflex sensitivity (BRS) is the body’s ability to adjust heart rate (HR) to control blood pressure. Traditionally, BRS is quantified by measuring HR changes (obtained via an electrocardiogram [ECG]) following alterations in arterial pressure (conventionally measured through an arterial line). However, the invasiveness of arterial line necessitates alternatives, such as the volume clamp method and the less invasive pulse photoplethysmography (PPG). Notably, the PPG method is also suitable for continuous and free-living conditions.

**Objective:**

This study aims to compare PPG-based features for BRS determination based on the volume clamp method and gold standard arterial line. Data from a previous study was used where the primary analysis focused on evaluating the accuracy of PPG-derived HR variability, while this analysis quantifies BRS by measuring HR changes following alterations in arterial line pressure or less invasive alternatives. In addition, we investigate the feasibility of assessing BRS patterns over 24 hours using data from a single volunteer.

**Methods:**

A total of 28 male volunteers (age 52, SD 7 y; BMI 27, SD 4 kg/m^2^) equipped with four sensing modalities: (1) arterial line [ABP], (2) infrared PPG, (3) volume clamp finger pressure (VCP), and (4) ECG, performed a protocol of 3 repetitive sessions in supine position. For the extended feasibility of continuous BRS measurement, ECG and PPG data were acquired for 24 hours in free-living conditions from a normotensive male volunteer (33 y). BRS index was calculated within the low-frequency window (0.04‐0.15 Hz) averaging over all trials for each intervention and participant. A transfer function was estimated with systolic blood pressure (SBP) or its surrogate as input and HR (from the ECG) as output.

**Results:**

PPG-based BRS features, specifically the rise-decay time ratio (RDRatio) and pulse arrival time (PAT), demonstrate intraparticipant precision of 44% and 23%, respectively, with interparticipant variation of 91% and 53%. The correlation of BRS_PAT,PPG_ and BRS_RDRatio,PPG_ with the gold standard BRS_SBP,ABP_ (SBP) is 0.66 and 0.56, respectively. During intervention, the correlations remain high for BRS_RDRatio,PPG_ (rest: 0.75, paced-breathing: 0.50, and handgrip: 0.46) and BRS_PAT,PPG_ (rest: 0.69, paced-breathing: 0.52, and handgrip: 0.62). In the 24-hour data, the BRS_PAT,PPG_ and BRS_RDRatio,PPG_ exhibit changes during the day corresponding to the activity levels and SBP variations. Notably, during the night, a cyclic rhythm is observed for both BRS_PAT,PPG_ and BRS_RDRatio,PPG_.

**Conclusions:**

This study demonstrates that in male volunteers, PPG-based PAT and RDRatio BRS serve as suitable surrogates for gold-standard BRS derived from arterial line, showing the highest correlation and comparable intraparticipant coefficient variation. Furthermore, they show expected changes during controlled activities and a 24-hour feasibility test in free-living conditions.

## Introduction

The baroreflex is a feedback system controlling arterial blood pressure (ABP). Stretch receptors in the aortic arch wall and carotid sinuses sense the changes in ABP. When arterial transmural pressure increases, the baroreflex responds by lowering heart rate (HR) and decreasing cardiac contractility and reducing peripheral vascular resistance, and vice versa [1].

Baroreflex sensitivity (BRS) refers to the body’s ability to adjust HR in response to changes in blood pressure (BP). Maintaining this hemodynamic homeostasis is a continuous process and of vital importance to healthy organ perfusion. A decrease in baroreflex sensitivity is associated with (persistent) hypertension, heart failure, poor outcome after stroke and kidney failure [2-8].

BRS can be modeled in both the time and frequency domain. Traditionally, it is quantified by measuring the HR changes following variations in arterial pressure. The sequence method is a time-domain method in which 3 or more consecutive beats with progressively increasing or decreasing arterial pressure are followed by a progressive increase or decrease of HR [9]. The frequency domain or spectral analysis is applied on continuous electrocardiogram (ECG) and arterial pressure signals assuming changes in arterial pressure and HR, induced by the baroreflex, are oscillations in the same frequency band. In the frequency domain analysis, different strategies are used, including nonparametric transfer function, parametric transfer function, and the phase rectified signal averaging method [1,7]. The most used BRS method calculates the spectral relationship between the input signal, commonly the systolic blood pressure (SBP) obtained from the continuous arterial pressure signal, and the output signal, commonly the RR interval (interbeat time interval based on R-peak of ECG) obtained from the ECG [7,10]. The BRS is typically quantified as the average spectral gain within the low frequency (LF; 0.04‐0.15 Hz) or high frequency (HF; 0.15‐0.40 Hz) window [7,8,10,11].

The gold standard for measuring arterial pressure is directly through an arterial line, that is, a (fluid-filled) catheter inserted into an artery. However, due to the invasiveness of this technique, alternative methodologies are necessary. A commonly used alternative is the continuous finger BP measured using the volume clamp method. This method uses a 2-sensor system that combines photoplethysmography (PPG) and a pressure sensor [12,13]. Previous investigations have revealed that, depending on the device, the variability of the systolic pressure has been overestimated by 78% and 103% in the low-frequency band [13]. In addition, the same investigation demonstrated an underestimation of the baroreflex sensitivity by −24% or −31%. Another, more indirect method to estimate ABP is through PPG, currently predominantly used in research settings [14]. PPG measures the blood volume pulse through light transmission instead of a direct pressure pulse. The PPG signal is composed of a pulsatile (“AC” [alternating current]) and baseline (“DC” [direct current]) part. The AC part reflects the changes in blood volume and is divided into a systolic phase (from foot to primary peak) and a diastolic phase (from secondary peak to foot) [15,16]. The PPG volume pulse contour is related to a pressure pulse [17]. Primary peak amplitude, referred to as systolic amplitude, has been related to stroke volume and changes under the influence of vasomotor tone and blood volume [15,16]. Different features have been proposed relating PPG with BP, including pulse arrival time (PAT), pulse width, reflection index, and PPG variability [18]. PAT is the time between the electrical activation of the left ventricle, obtained with ECG, and the arrival of the wave in the periphery. PAT is known to be related to the BP or SBP [14]. As BP increases, the apparent arterial stiffness increases and PAT decreases. Besides this inverse relation, PAT is also determined by the pre-ejection period, that is, the time between electrical activation and opening of the valve of the left ventricle. PPG has also been related to systemic vascular resistance and vasomotor tone. The DC component of the PPG and pulse width are determined by, among other things, the vasomotor tone [16,19-21]. Pre-ejection period change, related to change in cardiac contractility, can also be derived from the PPG signal [22,23]. Hence, PPG contains more baroreflex-related information than just arterial pressure. It has been used to determine BRS and is most often compared to BRS, based on the volume clamp method [10,24-26]. To the best of our knowledge, BRS obtained from invasive arterial pressure, volume clamp finger pressure (VCP), and PPG have not been compared directly.

Continuous BRS measurements in a free-living condition could elucidate the variation of the BRS and its potential interaction with the circadian rhythm. BRS over a 24-hour period is useful to monitor autonomic nervous system (dys)function at night in the absence of other influences, to relate it to sleep stages, for example, in patients with prediabetes [27], older adults [28], or patients with hypertension [29,30]. Long-term free-living monitoring requires a minimally obtrusive wearable solution, which could be PPG, for instance. To reliably use PPG for BRS determination, it is important to understand the benefits and the disputes compared to a direct BP measurement from the arterial line.

This study aims to better understand which PPG-based features for BRS determination perform best in comparison to BRS based on the volume clamp method and gold standard arterial line. In addition, this study, in extension, also aims to assess the feasibility of assessing BRS patterns over 24 hours by means of a single volunteer.

## Methods

### Overview

This study involves a secondary analysis of an existing dataset [31]. The primary analysis focused on evaluating the accuracy of PPG-derived HR variability (HRV) [31], while in this analysis, the baroreceptor sensitivity is quantified by measuring HR changes following alterations in arterial line pressure or less invasive alternatives.

### Datasets: Arterial Line Interventional Study

**Figure 1.**
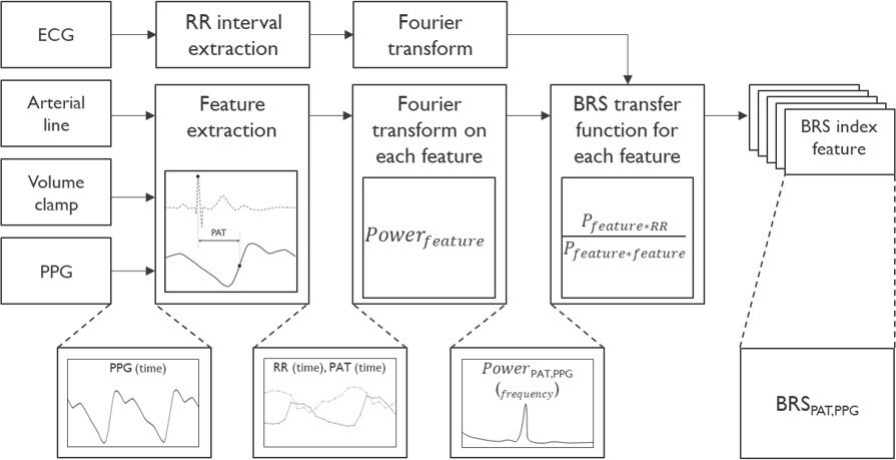
Overview of the steps to compute baroreceptor sensitivity (BRS) indices with example for pulse arrival time (PAT) based on photoplethysmography (PPG). ECG: electrocardiogram; RR: interbeat time interval based on R-peak of ECG.

The interventional dataset was used to analyze the differences and similarities between BRS derived from invasive arterial line BP, VCP, and PPG. More details can be found in [31]. In summary, 28 male healthy volunteers (aged 52, SD 7 y; BMI 27, SD 4 kg/m^2^, SBP 130, SD 12 mm Hg). Participants were equipped with four sensing modalities (see Figure 1): (1) arterial line inserted into radial artery on the nondominant arm, (2) infrared PPG at the index finger of the same arm (Biopac PPG100C, 240 Hz), (3) Finapress Nova at middle finger of the same arm, and (4) ECG in lead II configuration (Biopac ECG100C, 240 Hz). Participants performed a protocol of 3 repetitive sessions in supine position with the arm resting alongside the body. Each session included several interventions, namely two times paced breathing at 7 breaths for 3 minutes. A handgrip intervention during which the participant was asked to squeeze in a handgrip as much as possible for one and a half minutes using their dominant hand. In addition, a dedicated rest period where the participants were asked to close their eyes for 2 minutes. Extra (unlabeled) transition time was allocated in between activities.

### Ambulatory 24-Hour Study

In addition, a single 24-hour recording on a healthy volunteer was used to demonstrate BRS trends under free living conditions. In a normotensive male (33 y), a wearable prototype developed by imec was placed that recorded ECG and PPG for 24 hours. The ECG was placed in lead II configuration, and the transmissive PPG sensor Nonin 8000J was placed on the left index finger. In addition, an ambulatory BP measurement device (Suntech Medical Oscar2) recorded cuff-based oscillometric BP from the left upper arm in intervals of 15 and 30 minutes during the day and night, respectively. This was a regular office day, including 8 hours of sleep, 2 walks, and office work behind a desk.

### Ethical Considerations

The interventional study dataset was collected at Ziekenhuis Oost-Limburg in Genk, Belgium, and has been approved by the institutional review board (ethical committee approval 16/039U). All enrolled participants were compensated with a US $135 voucher. The ambulatory 24-hour feasibility data have been approved by the institutional review board of imec The Netherlands in Eindhoven, the Netherlands.

Both studies were conducted under the principles of the Declaration of Helsinki. All eligible participants were given the right to refuse participation and the right to withdraw from the study at any time. Written informed consent was collected from all participants before participation. To protect the participants’ privacy, all data collected from this study were kept confidential and anonymized and were only accessible to the members of the research team.

### Data Analysis and Statistics

#### Data Preprocessing

**Figure 2.**
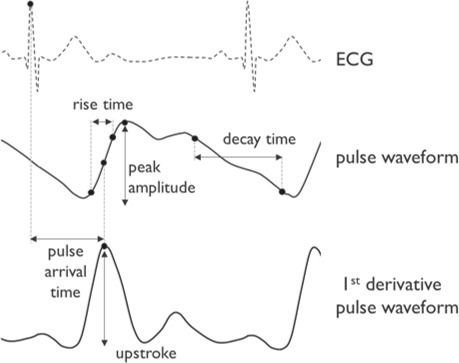
Visual representation of the features as described. Dashed line is the electrocardiogram (ECG) signal, the solid lines the pulse waveform from either photoplethysmography (PPG), volume clamp or arterial line, and first derivative of the PPG (PPG’).

Data was processed and analyzed using Matlab R2022a (Mathworks). The relevant features in the pulse waveforms were computed from fiducial points detected in the first derivative signal, as described in detail by Fedjajevs et al [32] (see Figure 2). In brief, all data was band-pass filtered using a Butterworth low-pass filter with the cutoff at 10 Hz and high-pass filter acting as a differentiator. The differentiated signal is used to first find the upstroke, the local maximum of the differentiated signal. Next, the other fiducial points—peak, foot, shoulder, secondary peak, and dicrotic notch—were extracted using adaptive thresholding. Timestamps of the fiducial points are used to calculate the rise and decay times, amplitudes, and their ratios.

#### Features

Features were computed per beat for all pulse waveforms, that is, PPG, volume clamp, and arterial line. Figure 2 shows a visual representation of the derived features. Peak amplitude (PA) is the amplitude of the first peak of the PPG. Pulse upstroke was defined as the amplitude of the peak in the first derivative of the PPG signal. Pulse arrival time (PAT) was defined as the delay between the R-peak from the ECG and the pulse upstroke in the PPG. Rise-Decay Ratio (RDRatio) was defined as the ratio between the rise time (10% to 70% of the peak amplitude in the systolic phase) and decay time (70% to 10% of the peak amplitude in the diastolic phase).

Volume clamp and arterial line features were derived in a similar manner as described for PPG and are indicated by the respective subscripts V (volume clamp) and A (ABP). SBP is the peak amplitude of either pressure pulse wave.

#### Transfer Function

BRS indices of various modalities were calculated in the low frequency window (0.04‐0.15 Hz) taking the average over all trials per intervention per participant. Data was resampled to 4 Hz, and a first-order trend was removed. A transfer function was estimated with feature (SBP or surrogate) as input and HR (RR interval derived from the ECG) as output. The transfer function was defined as the ratio of the cross power spectral density of the input (x, respectively BP) and output (y, respectively RR interval) and the spectral density of the input (x):


(1) $$H\left(f\right)=\frac{P_{xy}}{P_{xx}}=\frac{P_{feature*RR}}{P_{feature*feature}}$$

Coherence levels were determined between SBP (or a surrogate feature) and RR interval for each BRS index. Considering the number of unique data points per segment (120 data points with 50% overlap), the threshold coherence at 95% CI is set at 0.14 [33,34].

#### Analysis of Arterial Line Data

A structured analysis was done based on different BRS indices, calculated using the RR intervals from the ECG as input and as output the SBP from the arterial line (BRS_SBP,A_ as gold reference) or other features as potential surrogates. The analysis included the following steps: Step 1, the direct comparison of BRS based on the SBP for the arterial line (BRS_SBP,ABP_) and volume clamp (BRS_SBP,V_) and the corresponding feature peak amplitude of the PPG (BRS_PeakAmp,PPG_). Step 2, the comparison of various features (upstroke, PAT, and RDRatio) obtained from the arterial line sensor, as this is the signal that is the most direct BP recording with the highest signal to noise ratio. Followed by step 3, the comparison of BRS obtained from the same features (SBP or PeakAmp, upstroke, PAT, and RDRatio) from arterial line and PPG. Finally, step 4 a comparison of BRS based on different features (SBP or PeakAmp, pulse upstroke, PAT, and RDRatio) obtained from volume clamp and PPG with the gold reference, namely BRS obtained from arterial line SBP.

#### Statistical Analysis

For each participant and intervention, BRS measures were computed across all modalities and features. To mitigate the impact of outliers, we calculated the BRS index as the median value derived from three repeated measurements. The precision of each BRS index within each participant (intraparticipant precision) was determined by calculating the SD of the error. This error is the difference between the median of the participant and the individual values. The result was then expressed as a percentage of the mean BRS indices across all participants. Variation between participants (interparticipant variation) was calculated by determining the SD of the BRS indices. This was also expressed as a percentage of the mean BRS indices across all participants. In the final analysis, the correlation among the various BRS indices was determined using both Pearson and Spearman correlation methods, as not all BRS indices were normally distributed. It is important to note that the results from both the parametric (Pearson) and nonparametric (Spearman) methods were largely similar. Therefore, for simplicity, only the results derived from Pearson correlation analysis are reported. The level of statistical significance was set to .05.

#### Analysis 24-Hour Data

The 24-hour dataset was processed in the same way as the interventional dataset with additional windowing. Based on results from arterial line data, PAT and RDRatio were selected as the best option for 24-hour BRS features (see Results and Discussion). From the filtered continuous ECG and PPG data acquired by the wearable prototype, beat-to-beat RR intervals, PAT, and RDRatio were computed. Both raw signals and extracted features were (dis)qualified based on an integration of 5 objective criteria:

First, any beat-to-beat samples 15 seconds before and 60 seconds post the cuff inflation due to occlusion.

Second, beat segments in the PPG signal were qualified using a proprietary signal-to-noise ratio (SNR) algorithm wherein a reference signal template is defined by 5 consecutive beats, and the noise impacting the signal morphology (eg, due to motion) is defined as the deviation of individual beats from this template and disqualified by empirical thresholds.

Third, absolute thresholding of beat-to-beat PAT samples deviating from a physiologically valid range, under the assumption of a fixed distance and pulse wave velocities from 2 to 10 m/seconds.

Fourth, variability thresholding of drastic beat-to-beat changes in HR and PAT deviating from physiologically valid ranges of HRV and PAT variability (respectively sympathetic changes in BP and arterial stiffness).

Fifth, disqualification of 1-minute epochs of persistent low quality, wherein the more robust ECG signal defines the expected number of cardiac cycles, and at least 50% of the PPG beats ought to be present and not undetected or disqualified by the previous criteria.

From here, BRS_PAT,P_ and BRS_RDRatio,P_ were calculated over a 4-minute sliding window (75% overlap), whereafter the median was taken from all BRS values exceeding the coherence threshold of 0.14 within a 1-hour sliding window (75% overlap). Cuff-based BP was measured every 15 minutes during the day and every 30 minutes during the night, providing at least two reference BP measurements included within the 1-hour sliding window. BP readings were qualified for validity by proprietary software of the ambulatory BP monitor (Suntech Medical Oscar2). The participant was also asked to stand still during the cuff inflation, which ensures stable ECG and PPG signals from the wearable system. For all clarity, no BRS was computed from the ambulatory cuff BP due to overlong sampling intervals. The BRS coherence was calculated over 4-minute windows per feature, and the median coherence for the subsequently computed 1-hour window held only the samples above the coherence threshold. Furthermore, given the longitudinal character of the 24-hour dataset, HRV as an indicator of autonomous nervous system activity was computed for relevant frequency bands: high frequency HRV_HF_, reflecting the parasympathetic-driven respiratory band around 15 cycles per minute or 0.25 Hz on average, and the low frequency HRV_LF_, reflecting baroreflex activity (balanced by sympathetic & parasympathetic activity) around 6 cycles per minute or 0.1 Hz on average [35]. Consistent with other features, the HRV indices were also averaged with a 1-hour sliding window. Ultimately, for visual inspection, all 1-hour averaged features are displayed on a time grid of 15-minute intervals.

## Results

### Arterial Line Intervention Study

The dataset consisted of 28 male volunteers. Per participant, 3 sessions were recorded of several interventions, including the interventions analyzed here: rest, paced breathing, and handgrip. A total of 420 segments were analyzed to extract BRS values of different (surrogate) features. By averaging over the repeated measures, we obtained 140 data points for each participant and each intervention.

For all participants and interventions, the mean of the BRS_SBP,ABP_ and BRS_SBP,VCP_ were 8.1 (SD 3.0) milliseconds/mm Hg and 6.6 (SD 3.0) milliseconds/mm Hg, respectively, with intraparticipant precision of 15% for BRS_SBP,ABP_ and 21% for BRS_SBP,VCP_, respectively. The PPG-based features show mean values of 3.2 (SD 1.9) au for BRS_PeakAmp,PPG_, 93 (SD 60) au for BRS_Upstroke,PPG_, 1.8 (SD 1.7) milliseconds for BRS_RDRatio,PPG_, and 7.7 (SD 4.1) milliseconds/milliseconds for BRS_PAT,PPG_.

The intraparticipant precision for the PPG-based features is on average higher: 54% for BRS_PeakAmp,PPG_, 56% for BRS_Upstroke,PPG_, and 44% for BRS_RDRatio,PPG_. The intraparticipant precision of the BRS_PAT,PPG_ approaches that of the traditional BRS index (23% for BRS_PAT,PPG_, compared to 20% and 26% for BRS_SBP,ABP_ and BRS_SBP,VCP_, respectively). In all cases, the interparticipant variation (37% for BRS_SBP,ABP,_ 46% for BRS_SBP,VCP_, 59% for BRS_PeakAmp,PPG_, 64% for BRS_Upstroke,PPG_, 91% for BRS_RDRatio,PPG_, and 53% for BRS_PAT,PPG_) exceeded the intraparticipant precision.

As described in the methods section, analysis was done in a step-by-step approach.

#### Step 1

Direct comparison between the peak amplitude feature between sensor modalities showed that the correlation between BRS_SBP,ABP_ and BRS_SBP,VCP_ was 0.78. A lower correlation was found between the BRS_SBP,ABP_ and BRS_PeakAmp,PPG_ (*r*=0.59) and BRS_SBP,VCP_ and BRS_PeakAmp,PPG_ (*r*=0.56). These correlations were all highly significant. Note that the absolute values of the BRS_PeakAmp,PPG_ cannot be compared to those of the SBP-based BRS values as the unit is different.

#### Step 2

In Table 1, the correlation of BRS obtained from different features from the arterial line sensor showed that BRS_SBP,ABP_ varied from strong to moderate for the different surrogate features BRS_RDRatio,ABP_ (*r*=0.66), BRS_Upstroke,ABP_ (*r*=0.54), and BRS_PAT,ABP_ (*r*=0.46), all *P*<.05

**Table 1.** Correlation of baroreceptor sensitivity between alternative features derived from arterial line and systolic blood pressure from arterial line. Pearson’s correlation between baroreceptor sensitivity index (BRS) of features extracted from arterial line with respect to the gold-standard reference feature systolic blood pressure from arterial line (BRS_SBP,ABP_). For details about the features, see Figure 2 and the Methods section.

Arterial line features	Correlation
BRS_PAT,ABP_	0.46^a^
BRS_Upstroke,ABP_	0.54^a^
BRS_RDRatio,ABP_	0.66^a^

a Indicates significant correlation (*P*<.05). BRS_PAT,ABP_, BRS_Upstroke,ABP_, and BRS_RDRatio,ABP_ are BRS surrogate indices obtained from arterial derived features pulse arrival time, upstroke gradient, and rise time-decay time ratio, respectively.

#### Step 3

Comparing the same feature between arterial line and PPG sensor modalities revealed that for peak-amplitude and upstroke derived BRS indices, the correlation was moderate and weak, respectively. In contrast, the correlation between PAT and RDRatio derived BRS indices across the sensor modalities was strong (see Table 2).

**Table 2.** Correlation between baroreflex sensitivity (BRS) extracted from arterial line (ABP) and PPG. BRS from arterial line (subscript _ABP_) and photoplethysmography (PPG or subscript _PPG_) using different features namely, systolic blood pressure (SBP), peak amplitude (PeakAmp), pulse arrival time (PAT), pulse upstroke (Upstroke) and rise time-decay time ratio (RDRatio). For details about the features, see Figure 2 and the Methods section. The primary peak (PeakAmp) of arterial line data is the systolic blood pressure (SBP).

Arterial line	PPG^a^	Correlation (Arterial line vs PPG)
BRS_SBP,ABP_	BRS_PeakAmp,PPG_	0.59^b^
BRS_PAT,ABP_	BRS_PAT,PPG_	0.75^b^
BRS_Upstroke,ABP_	BRS_Upstroke,PPG_	0.29^b^
BRS_RDRatio,ABP_	BRS_RDRatio,PPG_	0.49^b^

a PPG: photoplethysmography.

b Indicates significant correlation (*P*<.05).

#### Step 4

**Figure 3.**
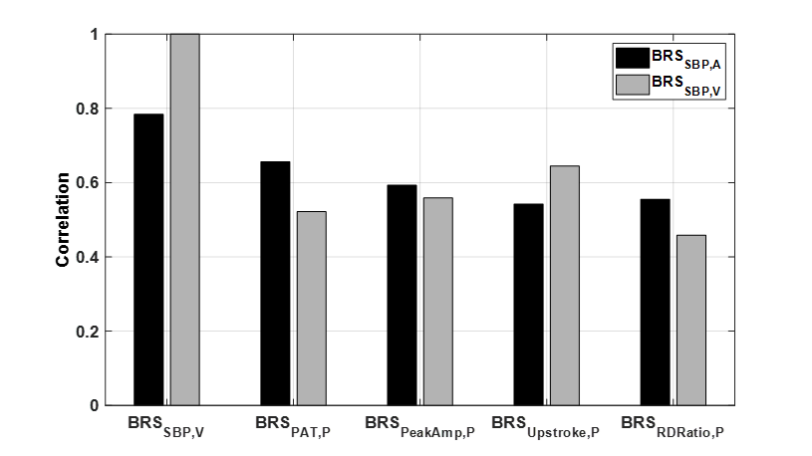
Correlation of baroreflex sensitivity (BRS) indices based on a selection of photoplethysmography (PPG) features with BRS from systolic blood pressure measured by arterial line (BRS_SBP,ABP_, black) and the volume clamp method (BRS_SBP,VCP_, gray). The BRS indices from PPG features BRS_PAT,PPG_, BRS_PeakAmp,PPG_, BRS_Upstroke,PPG_, and BRS_RDRatio,PPG_ are based on PPG and use pulse arrival time, peak amplitude, upstroke gradient, and rise time-decay time ratio, respectively. All correlations were significant (*P*<.05).

Figure 3 shows the comparison of features derived from the PPG, as target sensor, and arterial line SBP, as gold standard or volume clamp. All correlations in step 4 were significant. As expected, the best correlation was observed between BRS_SBP,ABP_ and BRS_SBP,VCP_ (*r*=0.78). The PPG-based BRS surrogates had a strong to moderate correlation with BRS_SBP,ABP_, where BRS_PAT,PPG_ (0.66) had slightly higher values compared to BRS_PeakAmp,PPG_, BRS_RDRatio,PPG_, and BRS_Upstroke,PPG_ (0.59, 0.56, and 0.54, respectively). However, when BRS_SBP,VCP_ was used as an alternative reference to BRS_SBP,ABP_ in the PPG-based surrogates that are based on PPG timing, a lower correlation was found with BRS_SBP,VCP_ with respect to BRS_SBP,ABP_ (0.52 vs 0.66 and 0.46 vs 0.56 for PAT and RDRatio, respectively). In contrast, the PPG amplitude derived parameters, that is, PeakAmp and upstroke had similar or even higher correlation with BRS_SBP,VCP_ compared to BRS_SBP,ABP_ (0.56 vs 0.59 and 0.64 vs 0.54, respectively; see Figure 3).

#### Interventions

**Figure 4.**
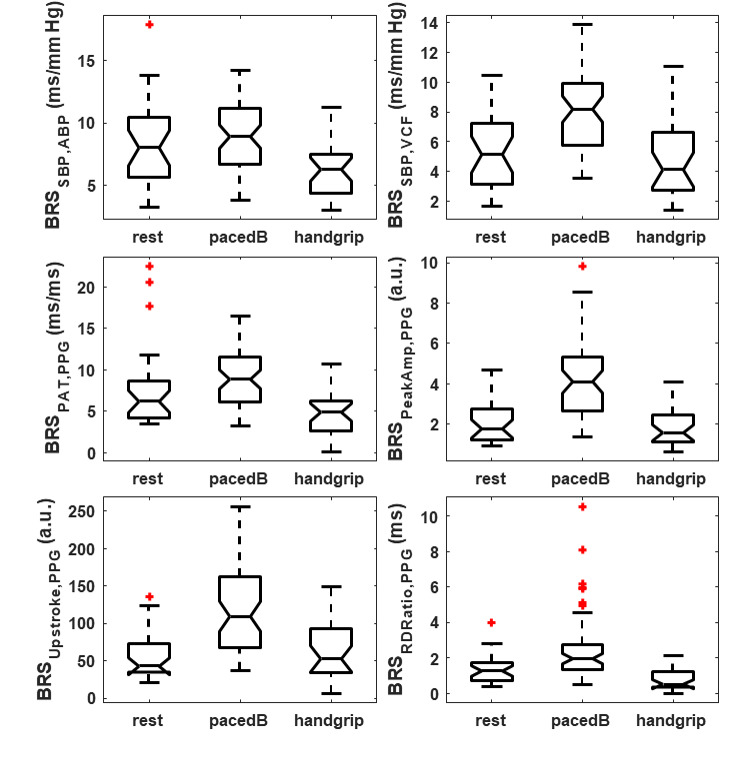
Baroreflex sensitivity (BRS) indices of different features for each intervention, namely: rest, paced breathing (pacedB), handgrip. BRS_SBP,ABP_, BRS_SBP,VCP_ are BRS indices based on systolic blood pressure measured by arterial line and volume clamp method, respectively. BRS_PAT,PPG_, BRS_PeakAmp,PPG_, BRS_Upstroke,PPG_, BRS_RDRatio,PPG_ are BRS indices measured using photoplethysmography using pulse arrival time, peak amplitude upstroke, and rise time-decay time ratio, respectively.

The gold-standard BRS, derived from arterial line SBP (BRS_SBP,ABP_), exhibited an 11% increase during paced breathing and a 22% decrease during handgrip, compared to rest (see Figure 4). A similar pattern was observed for BRS_SBP,VCP_ and BRS_PAT,PPG_. However, the increase in BRS during paced breathing was more pronounced for these indices (58% and 42%, respectively). The BRS_RDRatio,PPG_ showed a comparable increase (54%) during paced breathing as BRS_SBP,VCP_ and BRS_PAT,PPG_, but its reduction during handgrip was more significant (60%). The changes in BRS calculated from the other features BRS_PeakAmp,PPG_ and BRS_Upstroke,PPG_ were considerably larger during paced breathing with increases of 133% and 155% respectively. At rest, the correlation between BRS_SBP,ABP_ and both BRS_SBP,VCP_ and BRS_PAT,PPG_ was similar. However, during paced breathing, the correlation with BRS_SBP,ABP_ decreased for BRS_PAT,P_ but increased for BRS_SBP,VCP_ (see Table 3). The correlation between the other surrogate indices and the arterial line was generally lower across interventions, with the exception of BRS_RDRatio,PPG_, which showed a high correlation with BRS_SBP,ABP_ under rest conditions.

**Table 3.** Correlation between baroreflex sensitivity (BRS) extracted from volume clamp (VCP) and PPG and gold reference BRS based on arterial line derived systolic blood pressure (BRS_SBP,ABP_). BRS obtained from systolic blood pressure measured by arterial line (BRS_SBP,ABP_) is correlated to BRS obtained from SBP measured using volume clamp (BRS_SBP,VCP_). In addition, BRS from photoplethysmography (PPG) is obtained with pulse arrival time (PAT), peak amplitude (PeakAmp), pulse upstroke (Upstroke), and rise time-decay time ratio (RDRatio). Note that the primary peak (PeakAmp) of arterial line and volume clamp data is the systolic blood pressure. Interventions are rest, paced breathing (pacedB), and handgrip. For further details, see the Methods section.

Correlation to BRS_SBP,ABP_	Rest	PacedB	Handgrip
BRS_SBP,VCP_	0.66^a^	0.88^a^	0.78^a^
BRS_PAT,PPG_	0.69^a^	0.52^a^	0.62^a^
BRS_PeakAmp,PPG_	0.56^a^	0.51^a^	0.53^a^
BRS_Upstroke,PPG_	0.33	0.65^a^	0.16
BRS_RDRatio,PPG_	0.75^a^	0.50^a^	0.46^a^

a Indicates significant correlation (*P*<.05).

### Ambulatory 24-Hour Study

**Figure 5.**
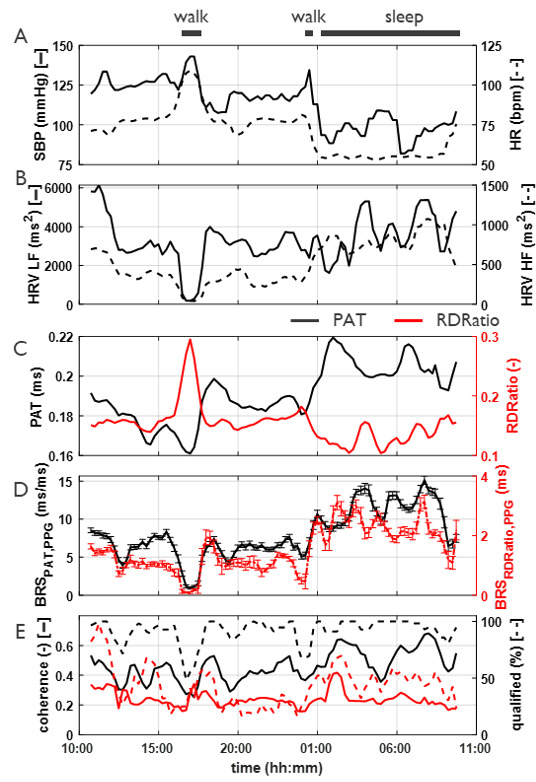
Analysis of the 24-hour trends for an individual participant. (A) trends of reference systolic blood pressure (based on upper arm cuff) and heart rate (HR) over time, (B) trends of low frequency (HRV LF) and high frequency heart rate variability (HRV HF). (C) Trends in the features pulse arrival time (PAT, in black) and rise time-decay time ratio (RDRatio, in red). (D) Trends in baroreflex sensitivity calculated from pulse arrival time (BRS_PAT,PPG_) and rise time-decay time ratio (BRS_RDRatio,PPG_). The error bars indicate the standard error of the BRS values within 15-minute segments. (E) Trends in coherence and qualified percentage of BRS_PAT,PPG_ and BRS_RDRatio,PPG_ values. The horizontal bars on top show the activities (walking and sleeping) of the individual.

Figure 5 shows the exploratory 24-hour BRS recording from a healthy participant in free-living conditions. Except for a 1-hour walk around 5 PM and a short walk before 12 AM, the participant spent most of the day doing sedentary work. The participant was in bed between 12 and 8 AM. The lowest BRS was observed in the afternoon around 5 PM when the participant went for a walk, as also indicated by relatively high HR and low HRV. The activities were self-reported.

The BRS indices show a clear pattern over the 24 hours, with both BRS_PAT,PPG_ and BRS_RDRatio,PPG_ being at a lower level during the day and increasing at night, effectively during sleep. Given the inverse relation between PAT and SBP, the mirrored pattern of increasing SBP and decreasing PAT (and vice versa) is clearly evident, also during walking activities with contributing HR. The correlation coefficients between PAT and RDRatio with SBP were high at −0.90 and −0.63 (both *P*≤.05), respectively throughout the 24-hour trajectory. This confirms the essential validity of the observed trends based on the processed and qualified data. Interestingly, the correlation between SBP with the derived BRS indices (BRS_PAT,PPG_ and BRS_RDRatio,PPG_) was equally high and significant (*r*=−0.74 vs *r*=−0.75; both *P*≤.05, respectively), while their mutual correlation was better (*r*=0.84; *P*≤.05). At a close look, the rhythmic oscillations during the night cannot only be seen in BRS_PAT,PPG_ and BRS_RDRatio,PPG_ but also in HRV_LF_. Unlike HRV_LF_, HRV_HF_ does not show any significant oscillations during the night, but instead displays a clear difference in absolute BRS level between day and night, which could not be observed in HRV_LF_. Coherence of BRS_PAT,PPG_ also shows a slight increase during the night. Except for a few datapoints during the walking activities, where the measurements were affected by motion artifacts, the percentage of BRS_PAT,PPG_ above the coherence quality threshold of 0.14 never dropped below 80%, while the qualified coherence % of BRS_RDRatio,PPG_ was significantly lower, as directly reflected in the coherence profile of BRS_RDRatio,PPG_. At all times, even during the walking events where motion artifacts are to be expected, sufficient data is above coherence threshold ensuring valid median values throughout the day.

## Discussion

### Principal Findings

This study illustrates that both the BRS based on PAT and the RDRatio derived from PPG serve as appropriate substitutes for the gold-standard BRS obtained from arterial lines. This is substantiated by the highest correlation observed during rest, a comparable coefficient of variation within participants, as well as anticipated alterations during activities. Furthermore, the feasibility of these measures was successfully tested over a 24-hour period under free-living conditions. This underscores their potential applicability in real-world scenarios.

### Evaluation of BRS Index Surrogates

Baroreflex sensitivity based on arterial line SBP is best correlated with BRS determined from pulse arrival time derived from PPG (PAT,P) signal: 66% of the variation in BRS_SBP,ABP_ is explained by BRS_PAT,PPG_. The correlation of other surrogates to BRS_SBP,ABP_ is slightly lower. In almost all cases, the interparticipant variation is higher than the intraparticipant precision, which suggests that these BRS features can be used to identify differences between individuals.

The baroreflex plays a crucial role in maintaining BP, acting through various pathways to modulate HR, vascular resistance, and cardiac contractility. The challenge in determining the BRS noninvasively is measuring (systolic) arterial pressure as the gold-standard method, arterial line, is invasive or obtrusive. The volume clamp method is an often-used noninvasive substitute to determine arterial pressure. It is known to overestimate the central ABP [36] and shown to overestimate BRS in the low frequencies [37]. Nevertheless, it has been used as a reference to validate other BRS methods, like those derived from PPG [10,24-26]. Comparing BRS using arterial pressure derived from the invasive arterial line with volume clamp method and PPG reveals the disputes and benefits of the methods. The BRS based on SBP derived from the volume clamp method shows the best correlation of 0.78 with BRS based on SBP from the invasive arterial line and a correlation of 0.59 with the BRS based on the systolic peak in the PPG signal. The latter is lower compared to results from others, reporting a correlation of 0.77 [10]. Nevertheless, our study population is considerably older (compared to 28.5 y) and has relatively high BP, which would lower the BRS and, in turn, increase the influence of noise, thereby reducing the correlation. An underestimation of the BRS based on SBP from the volume clamp method compared to the arterial line–based SBP of 24% was reported [13], similar to the 19% reported here.

The 3 modalities, arterial line, volume clamp, and PPG, have differences and similarities important to consider. The volume clamp method uses a PPG signal as well. Although the PPG signal is not used to measure the arterial pressure directly, it is used to maintain a constant volume by adjusting the cuff pressure, such that it equals the finger arterial pressure. Therefore, in contrast to the arterial line method, both PPG and volume clamp methods are influenced by peripheral perfusion and, hence, temperature, motion, and contact pressure. This could potentially cause errors when relating peripheral to systemic hemodynamic changes [15]. These errors would be visible between arterial line and volume clamp or PPG-derived features but be similarly present between PPG and volume clamp–derived features.

The range of BRS features derived from the PPG used here is also reported previously, like pulse upstroke, peak amplitude, pulse arrival time, and rise time [10,24]. In addition, models estimating SBP using PPG use PAT as the most important feature [14]. Pulse arrival time and rise-decay ratio from PPG correlate equally well with BRS_SBP,ABP_ and BRS_SBP,VCP_. Primary peak and pulse upstroke from PPG correlate well with BRS_SBP,VCP_, but notably less with BRS_SBP,ABP_, suggesting volume clamp underestimates the performance of BRS_PAT,P_ and BRS_RdRatio,P_ and overestimates the performance of others like BRS_Upstroke,P_. These results suggest that BRS_SBP,V_ can be used as a method to measure BRS in a noninvasive way; however, care should be taken to check the performance of other (PPG-derived) BRS indices using this method.

Coherence was used as a measure of BRS reliability. It assesses the similarity of two signals in the frequency domain; if the HR and arterial pressure have similar frequency components as a result of the baroreflex, the BRS becomes more reliable.

BRS changes from rest to controlled activities show how well a feature tracks the baroreflex effects of the interventions. The correlation between SBP and HR increases for paced breathing intervention; therefore, an increase in BRS during paced breathing is expected. Especially at 6 breaths per minute, at which the HR and BP oscillation amplitude are increased [38]. Expectedly, during paced breathing, BRS increases for BRS_SBP,ABP_ and all BRS surrogates compared to that at rest. However, the correlation between BRS_SBP,ABP_ and BRS_PAT,PPG_ decreases with paced breathing. It has been shown that during slow breathing of around 6 breaths/minute the BRS is overestimated, since, in this case, other mechanisms in which respiration influences HR also concentrate in the LF frequency band [39]. During the handgrip intervention, the coherence is increased, likely because of a thoracic pressure increase damping the oscillatory pressure effect of respiration (data not shown). An overestimation of the BRS changes based on volume clamp and PPG compared to BRS_SBP,ABP_ is found, which is more prominent during paced breathing compared to handgrip or handgrip recovery.

### Ambulatory 24-Hour Study

The adequate robustness found for the BRS surrogates during controlled activities suggests a wider applicability for BRS monitoring, which was further assessed by means of a 24-hour recording under free-living conditions. Based on the structured analysis, the BRS based on PAT and RDRatio was considered the most promising to test for the 24-hour ambulatory; it had the lowest intraparticipant variation and highest correlation with BRS_SBP,ABP_ during rest.

A wearable prototype for continuous ECG and PPG signal acquisition allowed for computation of beat-to-beat PAT, RDRatio, and RR intervals, and thereby enabled the observation of characteristic patterns in BRS_PAT,PPG_, BRS_RDRatio,PPG_, and HRV.

The BRS indices were found to be higher during the night as compared to daytime, which is in line with previous experiments where BRS was obtained from an arterial line [28,40,41]. This expected behavior of increasing nighttime BRS (and a coherence up to 0.6) could be explained by the absence of other inhibitory influences on the baroreflex like emotional behavior and somatic afferent influences stimulated by muscle contraction, as proposed by [40].

In preceding 24-hour BRS studies, oscillatory patterns of BRS during nighttime were less prominent [28,30,40], likely because these studies either average over participants or longer time windows and typically report one datapoint per hour (unlike the 15 min interval in this study). Meanwhile, studies focusing on sleep stages do show an increase in baseline BRS during the night and oscillations in BRS and HRV_LF_ between rapid eye movement (REM) and non-REM sleep stages [42]. Supported by the findings from the interventional study and the coherence with HRV_LF_, the observed patterns in the proposed BRS indices BRS_PAT,PPG_ and BRS_RDRatio,PPG_ undoubtedly reflect nocturnal BRS oscillations. Furthermore, the frequency of oscillations also appears to be in line with the typical duration and cycle times of adult sleep stages [43]. However, this remains to be further investigated with proper reference technology and in a larger population.

Furthermore, the proposed BRS indices may add value beyond existing HRV metrics. That is, both BRS indices show the baseline increase and nocturnal oscillations, whereas HRV_LF_ does not show a clear baseline increase and HRV_HF_ does not show oscillations, and its baseline increase may also be driven by different nocturnal respiration levels. Meanwhile, oscillations of BRS_PAT,PPG_ tend to compare higher with HRV_LF_; yet, BRS_RDRatio,PPG_ seems to be more indicative of higher frequency contributions.

Regarding the reliability of the proposed indices, BRS_PAT,PPG_ shows constantly high coherence throughout the 24 hours. Even during activity, a sufficient percentage of qualified samples is present, which may be further enhanced with basic signal and feature qualification strategies. Although the coherence of BRS_RDRatio,PPG_ is substantially lower, it also remains above the threshold constantly with sufficient qualified samples. In terms of usability and technology integration, this may become a relevant compromise given that BRS_RDRatio,PPG_ holds the theoretical advantage to be computed using a (peripheral) PPG, hence without an ECG.

### Clinical Implications

The nature of the PPG sensor also allows for free-living recordings, enabling the monitoring of the BRS of the patients on day-to-day activities. The obtained results from the 24-hour study are encouraging future research, considering the wide range of clinical applications for longitudinal BRS monitoring: as a prognostic tool for heart attacks and arrhythmias not only as single point measurement but also during sleep [11], and for cardiac mortality in patients with renal failure [44], or as a predictor of outcome after surgery [45]. Furthermore, present knowledge may be enhanced in day-to-day assessment of spontaneous BRS, which previously relied on 8-minute recordings on two consecutive days [46]. Variations in the 24-hour recordings between young and elderly people have also been reported [28]. Establishing a 24-hour recording could therefore show not only the BRS in short BP changes but also on circadian BP patterns. Ultimately, longitudinal BRS monitoring bears large potential for hypertension diagnostics, in particular for primary hypertension whose origin is widely unknown.

### Limitations

The study on arterial lines does present certain limitations, primarily due to the relatively limited sample size and the inclusion of only male participants. Further research is required to examine the impact of factors such as age and arterial stiffness on the BRS indices, as well as to explore their interrelationships. Such comprehensive analysis necessitates the involvement of larger and more diverse cohorts. Overall, these preliminary patterns of the BRS_PAT,PPG_ and BRS_RDRatio,PPG_ over a 24-hour period under free-living conditions support the findings from the controlled interventional study, demonstrating that it is possible to use PAT and RDRatio derived from the PPG signal to estimate the BRS. However, the key limitation of the 24-hour study is the confinement to a single participant, but the findings give rise to further expand this study to investigate circadian and nocturnal BRS patterns in both healthy participants, and given the clinical relevance, also patient cohorts.

### Conclusions

BRS determined from pulse arrival time or RDRatio in a PPG signal is best correlated with BRS based on arterial line SBP. The BRS_PAT,PPG_ and BRS_RDRatio,PPG_ also follow the BRS_SBP,ABP_ during different physical activities. Furthermore, it allows for 24-hour BRS recordings, in which the expected circadian rhythm patterns are present.

## Abbreviations

ABP: arterial line blood pressure
AC: alternating current
BP: blood pressure
BRS: baroreceptor sensitivity
DC: direct current
ECG: electrocardiogram
HF: high frequency
HR: heart rate
HRV: heart rate variability
LF: low frequency
PA: peak amplitude
PAT: pulse arrival time
PPG: photoplethysmography
RDRatio: rise-time decay-time ratio
REM: rapid eye movement
RR interval: interbeat time interval based on R-peak of ECG
SBP: systolic blood pressure
SNR: signal to noise ratio
VCP: volume clamp finger pressure
